# Assessing the Utility of End-Tidal Carbon Dioxide as a Marker for Fluid Responsiveness in Cardiogenic Shock

**DOI:** 10.7759/cureus.13164

**Published:** 2021-02-05

**Authors:** Komal Baloch, Aziz Rehman Memon, Urwah Ikhlaq, Madiha Umair, Muhammad Imran Ansari, Jawed Abubaker, Nawal Salahuddin

**Affiliations:** 1 Critical Care Medicine, National Institute of Cardiovascular Diseases (NICVD), Karachi, PAK; 2 Internal Medicine, National Institute of Cardiovascular Diseases (NICVD), Karachi, PAK

**Keywords:** etco2, fluid responsiveness, cardiogenic shock, passive leg raise

## Abstract

Background

Preventing end-organ failure in patients with shock requires rapid and easily accessible measurements of fluid responsiveness. Unlike septic shock, not all patients in cardiogenic shock are preload responsive. We conducted this study to determine the discriminant power of changes in end-tidal carbon dioxide (ETCO_2_), systolic blood pressure (SBP), inferior vena cava (IVC) collapsibility index (IVC-CI), and venous to arterial carbon dioxide (Pv-aCO_2_) gap after a fluid challenge and compared it to increases in cardiac output.

Methodology

In a prospective, quasi-experimental design, mechanically ventilated patients in cardiogenic shock were assessed for fluid responsiveness by comparing improvement in cardiac output (velocity time integral) with changes in ETCO_2_, heart rate, SBP, Pv-aCO_2_ gap, IVC-CI after a fluid challenge (a crystalloid bolus or passive leg raise).

Results

Out of 60 patients, with mean age 61.3 ± 14.8 years, mean acute physiology and chronic health evaluation (APACHE) score ­14.82 ± 7.49, and median ejection fraction (EF) 25% (25-35), 36.7% (22) had non ST-segment elevation myocardial infarction (NSTEMI) and 60% (36) were ST-segment elevation myocardial infarction (STEMI). ETCO_2_ was the best predictor of fluid responsiveness; area under the curve (AUC) 0.705 (95% confidence interval (CI) 0.57-0.83), p=0.007, followed by reduction in Pv-aCO_2_ gap; AUC 0.598 (95% CI; 0.45-0.74), p= 0.202. Changes in SBP, mean arterial pressure (MAP), IVC-CI weren’t significant; 0.431 (p=0.367), 0.437 (p=0.410), 0.569 (p=0.367) respectively. The discriminant value identified for ETCO_2_ was more than equal to 2 mmHg, with sensitivity 58.6%, specificity 80.7%, positive predictive value 73.9% [95% CI; 56.5% to 86.1%], negative predictive value 69.7% [95% CI; 56.7% to 76.9%].

Conclusions

Change in ETCO_2_ is a useful bedside test to predict fluid responsiveness in cardiogenic shock.

## Introduction

Cardiogenic shock (CS) is a vicious cycle of cardiac injury and systemic decompensation associated with high morbidity and mortality. About 7-10% of patients with acute myocardial infarction are in cardiogenic shock on presentation [1]. The cascade involves declining stroke output leading to end-organ hypoperfusion, resulting in multisystem organ failure and death. Management strategies are based on early revascularization and hemodynamic support. Supportive measures include an assessment of the adequacy of preload [2]. Determining whether an increase in preload will improve cardiac function is the concept of ‘fluid responsiveness’.

Determining fluid responsiveness is now the standard of care for the management of shock due to any etiology [3]. In cardiogenic shock, compromised cardiac output can lead to excessive build-up of preload by blind and well-intentioned fluid boluses. This may actually worsen the cardiac output of a failing heart leading to progressive pulmonary edema and further deterioration of hemodynamic parameters [4]. Most of the studies about determining preload responsiveness have been done in septic shock, and our understanding of fluid responsiveness comes from them [5]. Hence the need for assessing fluid responsiveness in cardiac patients becomes imperative.

Traditional, static methods to assess fluid responsiveness like central venous pressure (CVP) and pulmonary artery occlusion pressures (PAOP) have lost their applicability in resuscitation protocols and can be unreliable in cardiogenic shock [6]. Dynamic indices such as stroke volume variation (SVV) and pulse pressure variation (PVV) are useful for assessing fluid responsiveness in septic shock but are invasive, require patients to be in sinus rhythm and on a set tidal volume of 8 ml/kg/I [7]. Hence, the utility of these methods is limited in cardiogenic shock, which frequently has patients with atrioventricular (AV)-nodal dysfunction and arrhythmias.

Simple bedside maneuvers like passive leg raise (PLR), capillary refill time (CRT), and end-tidal carbon dioxide (ETCO_2_) provide opportunities to make an assessment of fluid responsiveness in a patient easy and reliable, and of recent been the center of attention for physicians managing critically ill patients [8]. PLR is a well-described method to noninvasively provide a fluid challenge and thereby detect fluid responsiveness. The legs of a supine patient are raised to 45 degrees and held there for 30 to 90 seconds to assess a response [9]. Capnography is considered a simple, inexpensive, and non-invasive method to detect effective restoration of spontaneous circulation during cardiac arrest and estimate shock intensity in the early stage [10]. ETCO_2_ is known to be decreased in volume-related hypotensive states and has a correlation with blood pressure, serum lactate, and base excess [11,12]. A study conducted by Toupin et al. in general ICU patients reported that an increase of 2 mmHg in ETCO_2_ was associated with fluid responsiveness with a sensitivity of 75%, a specificity of 70%, and an area under the curve of 0.8 (95% CI, 0.7 to 0.9; P < 0.01) [13]. Monitoring of ETCO_2_ can be a good alternative to assess the expected increase in cardiac output after a fluid challenge.

We hypothesized that changes in ETCO_2_ after a fluid challenge to identify fluid responsiveness could be extended to patients with cardiogenic shock. The objective of our study was to determine the predictive ability of ETCO_2_ as a marker for fluid responsiveness in this group of patients.

## Materials and methods

We carried out a quasi-experimental study at a cardiology-dedicated, tertiary care teaching hospital located in a major urban center. The study duration was from January 2020 to June 2020. Consecutive adult patients who were mechanically ventilated, sedated, and in cardiogenic shock were included. Cardiogenic shock was defined as ejection fraction < 40% by echocardiogram, systolic blood pressure (SBP) <100 mmHg despite adequate fluid resuscitation (at least one liter of crystalloids or 500 mL of colloids and evidence of end‐organ damage (mottled skin, urine output <0.5 mL/kg for one hour, or arterial blood gas lactate more than 2 mmol/L) [14]. Patients with temporary pacemakers or atrial and ventricular arrhythmias were excluded.

To measure cardiac output, velocity time integral (VTI) was measured via echocardiography (Aplio i-series, i600; Canon Medical Systems Corporation, Ōtawara, Japan) by calculating blood flow across the aortic valve. Left ventricular outflow tract (LVOT)‐VTI was obtained by placing a pulsed‐wave Doppler sample volume in the LVOT immediately proximal to the aortic valve in the anteriorly angulated apical four‐chamber view and tracing the outer boundaries of the peak spectral Doppler signal [15]. Continuous end-tidal CO_2_ monitoring was obtained using Nihon Kohden CO_2_ Sensor TG-900P JG-900P (Nihon Kohden Corporation, Tokyo, Japan).

At baseline, ETCO_2_, VTI, SBP, mean arterial pressure (MAP), inferior vena cava (IVC) collapsibility index, and arterio-venous CO_2_ gap (Pv-aCO_2_ gap) from simultaneous arterial and venous blood gas were measured. A 10% increase in VTI was considered as an increase in cardiac output [16]. ETCO_2_ changes and the changes in other hemodynamic variables post fluid challenge were noted.

The managing team was then advised a fluid challenge given to the patients either as an auto-transfusion by passive leg raise (PLR) maneuver or a crystalloid bolus of 300 cc. The fluid bolus was administered to patients in whom a PLR maneuver was contraindicated, e.g., patients with an intra-aortic balloon pump (IABP). During PLR, the patient’s legs were lifted from a recumbent position to 45º with the help of two nursing staff for two minutes [17]. Patients were divided into two groups according to their response to fluid challenge as “fluid responders” and “fluid non-responders” as per a 10% rise in VTI from baseline.

Ethical approval for the study was obtained from the National Institute of Cardiovascular Diseases Ethical Review Committee (Approval number: 58/2019). Verbal informed consent for participation and publication was taken from the next of kin. The study has been performed in accordance with the ethical standards laid down in the 1964 Declaration of Helsinki and its later amendments. No individual data is presented.

Continuous variables are reported as means with standard deviation, and skewed distributions are reported as medians with interquartile ranges. Categorical variables are reported as proportions. Student t-test and chi-square tests were used to compare “fluid responders” with “fluid non-responders”. A receiver operating characteristic (ROC) curve was constructed comparing velocity time integral against changes in ETCO_2_, SBP, Pv-aCO_2_ gap, heart rate, and IVC collapsibility index. The area under the curve (AUC) along with its 95% confidence interval (CI) were obtained. Sensitivity (%), specificity (%), positive predictive values (PPV %), negative predictive values (NPV %), and accuracy (%) was calculated. A p-value <0.05 was considered statistically significant. IBM statistical package for the social sciences (SPSS) version 21.0 (IBM Corp., Armonk, NY) was used for all analyses.

## Results

Sixty patients in cardiogenic shock were assessed for fluid responsiveness. ­­­­­­Mean age was ­­­­­­61.3 ± 14.8 years and­­­­­ 40% (24) ­were females. About 53.3% (32) were known diabetics, 45% (27) were hypertensive, and 28.3% (17) had chronic obstructive pulmonary disease (COPD). Mean acute physiology and chronic health evaluation (APACHE) II score was 14.82 ± 7.49; 21 (35%) patients were on an intra-aortic balloon pump. Forty-four patients (73%) had acute kidney injury (AKI). Out of the 60 patients enrolled, 36.7% (22) presented with non ST-segment elevation myocardial infarction (NSTEMI) and 60% (36) with ST-segment elevation myocardial infarction (STEMI); 52.8% (19) had an anterior wall myocardial infarction. Mean ejection fraction was 25% [35% - 25%]. All patients were well sedated to maintain a constant minute ventilation. All were on vasopressors with doses titrated to a mean arterial pressure of ≥ 65mmHg (Table 1).

**Table 1:** Demographic and clinical characteristics of patients COPD = chronic obstructive pulmonary disease, STEMI = ST-segment elevation myocardial infarction, NSTEMI = Non ST-segment elevation myocardial infarction, Non-ICMP = non-ischemic cardiomyopathy, APACHE = acute physiology and chronic health evaluation.

Characteristics	Total
N	60
Gender	
Male	60% (36)
Female	40% (24)
Age (years)	61.3 ± 14.8
≤ 60 years	45% (27)
> 60 years	55% (33)
Risk factors	
COPD	28.3% (17)
Congestive heart failure (CHF)	9.8% (4)
Smoking	26.7% (16)
Diabetes	53.3% (32)
Hypertension	45% (27)
Chronic kidney diseases (CKD)	15% (9)
Ejection fraction (EF)	25 [35 - 25]
APACHE	14.82 ± 7.49
Myocardial infarction	
STEMI	60% (36)
Anterior Wall Myocardial Infarction	52.8% (19)
Inferior Wall Myocardial Infarction	19.4% (7)
Inferior Posterior Myocardial Infarction	11.1% (4)
Antero Inferior Myocardial Infarction	8.3% (3)
High Lateral Myocardial Infarction	5.6% (2)
Infero-Lateral Myocardial Infarction	2.8% (1)
NSTEMI	36.7% (22)
Non-ICMP	3.3% (2)
Acute kidney injury (AKI)	73.3% (44)
Intra-aortic balloon pump (IABP)	35% (21)

Out of 60 patients, 29 (48.3%) were fluid responders. There were no significant differences between the two groups concerning ejection fraction (p = 0.62) or the type of myocardial infarction (STEMI, p = 0.399; NSTEMI, p = 0.381). However, amongst the different types of STEMI, inferior wall myocardial infarction was found to be the most responsive to fluids, 0% (zero) non responders, 36.8% (seven) responders, p = 0.041 (Table 2).

**Table 2:** Fluid responders/non-responders in different types of myocardial infarctions PLR = passive leg raise, VTI = velocity time integral, STEMI = ST-segment elevation myocardial infarction, NSTEMI = Non ST-segment elevation myocardial infarction, Non-ICMP = non-ischemic cardiomyopathy, MI = myocardial infarction. *significant at 5%

	Fluid Responsiveness after PLR		P-value
	Non responders (∆VTI≤10%)	Responders (∆VTI>10%)	
Total (N)	31	29	-
Ejection fraction (EF)			
≤ 25%	58.1% (18)	51.7% (15)	0.622
> 25%	41.9% (13)	48.3% (14)	
Myocardial infarction			
STEMI	54.8% (17)	65.5% (19)	0.399
Anterior Wall MI	70.6% (12)	36.8% (7)	0.041*
Inferior Wall MI	0% (0)	36.8% (7)	
Inferior Posterior Wall MI	11.8% (2)	10.5% (2)	
Antero Inferior Wall MI	5.9% (1)	10.5% (2)	
High Lateral Wall MI	11.8% (2)	0% (0)	
Infero-Lateral Wall MI	0% (0)	5.3% (1)	
NSTEMI	41.9% (13)	31% (9)	0.381
Non-ICMP	3.2% (1)	3.4% (1)	0.962

**Figure 1:**
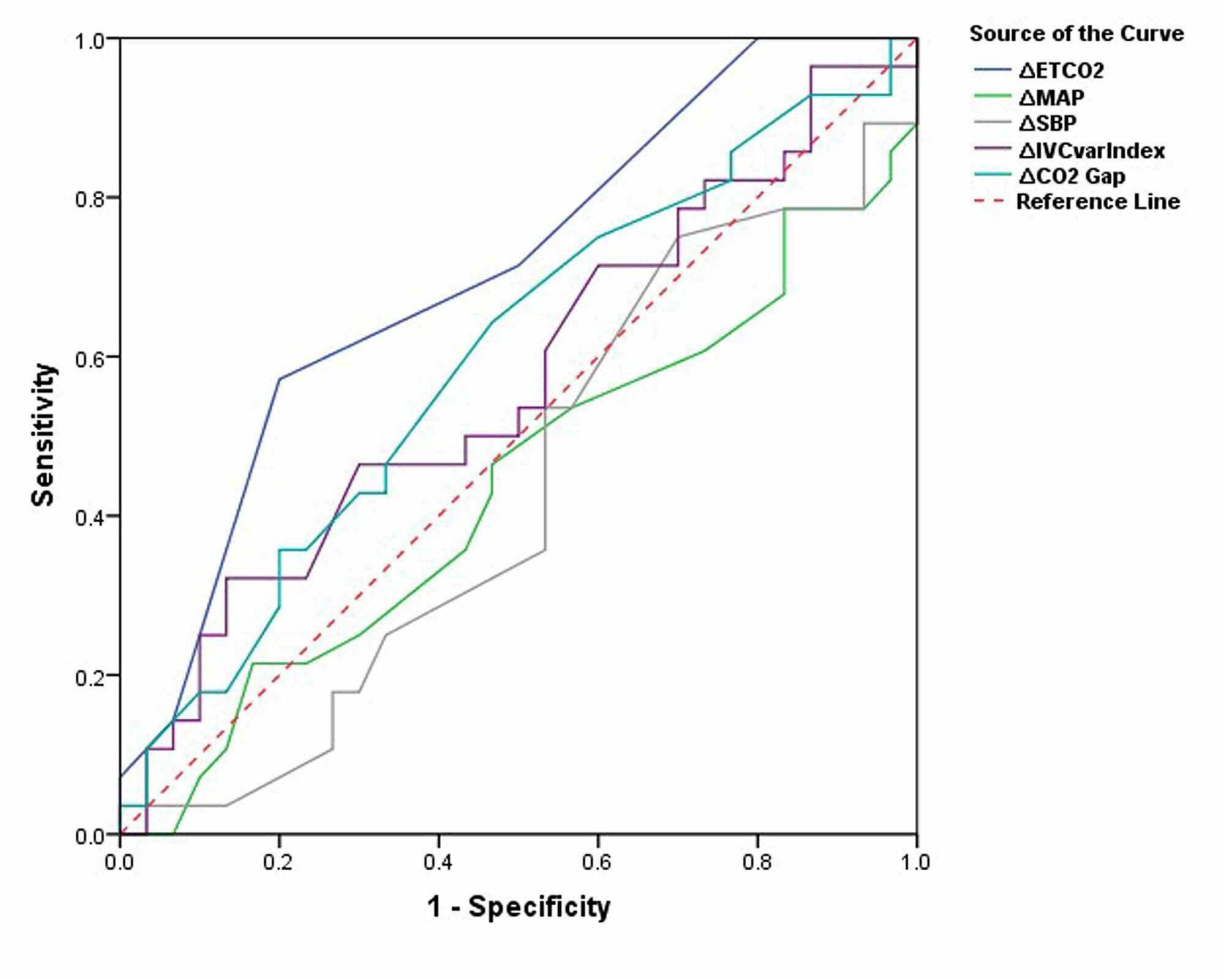
Receiver operating characteristic (ROC) curve analysis for fluid responsiveness ∆ETCO2 = change in end-tidal CO2, ∆MAP = change in mean arterial pressure, ∆SBP = change in systolic blood pressure, ∆IVCvarIndex = change in inferior vena cava collapsibility index, ∆CO2 Gap = change in partial pressure arterial-venous CO2

ROC curves showed an AUC of 0.705 (95% CI: 0.57, 0.83; p = 0.007) for change in ETCO2, 0.437 (95% CI: 0.28, 0.58; p = 0.410) for change in MAP, 0.431 (95% CI: 0.28, 0.58; p = 0.367) for change in SBP, 0.569 (95% CI: 0.42, 0.71; p = 0.36) for change in IVC collapsibility index, and 0.598 (95% CI: 0.45,0.74; p = 0.202) for change in Pv-aCO2 gap. Using the Youden index, a change in ETCO2 of two units was found to have a discriminant value for fluid responsiveness with a sensitivity of 58.6% [95% CI; 38.9%, 76.5%] and specificity 51.6% [95% CI; 33.1%, 69.9%] to detect fluid responsiveness (Table 3, Figure 1).

**Table 3:** Sensitivity analysis of change in ETCO2 > 2 as a diagnostic test for fluid response in cardiogenic shock VTI = velocity time integral, ∆ETCO2 = change in end-tidal CO2

	Fluid response as per VTI		Total
	Non responders (∆VTI≤10%)	Responders (∆VTI>10%)	
Fluid response as per ETCO2			
Non responders (∆ETCO2<2)	25 (41.7%)	12 (20%)	37 (61.7%)
Responders (∆ETCO2≥2)	6 (10%)	17 (28.3%)	23 (38.3%)
Total	31 (51.7%)	29 (48.3%)	60 (100%)
Accuracy	70.0% [95% CI; 56.8% to 81.2%]		
Sensitivity	58.6% [95% CI; 38.9% to 76.5%]		
Specificity	80.7% [95% CI; 62.5% to 92.5%]		
Positive Predictive Value	73.9% [95% CI; 56.5% to 86.1%]		
Negative Predictive Value	69.7% [95% CI; 56.7% to 76.9%]		

## Discussion

Our study revealed that changes in ETCO_2_ can dynamically identify improvements in cardiac output at the bedside after a fluid challenge and thereby provide a good bedside assessment of fluid responsiveness in patients with cardiogenic shock.

ETCO_2_ had been studied in the recent past for its useful correlation with cardiac output [18-20]. The simple principle behind its usage is based on its ability to detect changes in cardiac output (i.e., systemic perfusion) by changes in exhaled carbon dioxide when the minute ventilation and metabolism remains constant. Various studies have looked into the utility of ETCO_2_ as a marker of fluid responsiveness in septic shock patients [21,22]. Its use has been well documented in stable cardiac surgery patients to track changes in the cardiac index after PLR [13].

The management of shock is centered on optimizing oxygen delivery to the tissues [23]. It is relatively straightforward in patients with a septic shock where fluids become part and parcel of resuscitation. A cardiogenic shock, on the other hand, is not that straightforward. Usual care includes preload and afterload reduction and increasing cardiac contractility by using inotropes [3].

According to Frank-Starling mechanics, increasing preload by administering fluids can augment cardiac output if the heart is functioning at the steep portion of the volume-pressure curve. Measures to directly assess cardiac output non-invasively, like VTI and transthoracic electrical bio-impedance, or invasively by Swan-Ganz catheters or pulse contour analysis (PiCCO) or indicator dye dilution (LiDCO) are all standard of care but can be cumbersome and require expertise to assimilate information. There is a need for a simple and inexpensive bedside tool to follow changes in cardiac output during resuscitation of patients in shock, something that end-tidal CO_2_ monitoring fulfills.

Our study was different from others because our cohort were patients with cardiogenic shock. Giving too much or too little preload can either subject a patient to worsening pulmonary edema or end-organ hypoperfusion. We found that an increase in end-tidal CO_2_ of two has a sensitivity of 60% and specificity of 84% to predict fluid responsiveness. In a handful of studies in general ICU patients, a change in ETCO_2_ of two was found to predicate volume responsiveness [13,24-29]. Toupin et al. showed that in cardiac surgery patients, an increase of 2 mmHg in ETCO_2_ was associated with fluid responsiveness with a sensitivity of 75% and an AUC of 0.8 [13].

Change in ETCO_2_ is a direct reflection of cardiac output when minute ventilation and CO_2_ production are constant [26]. To address this, our patients were on sedation to achieve a constant minute ventilation.

The other indices compared with VTI in our study, such as changes in SBP, MAP, IVC collapsibility index, and Pv-aCO_2_ gap, were not found to be useful in predicting response to fluid. These results are consistent with those reported by other investigators. We hypothesize that the widespread sympathetic activation with a consequent increase in systemic vascular resistance may prevent a change in MAP or SBP with changes in cardiac output.

The main strength of our study is our study population comprised of patients in cardiogenic shock. The lack of data in this cohort regarding the key element of resuscitation, i.e., fluid, is a major reason we conducted this study. We evaluated a diverse variety of patients, including STEMIs (encompassing all the different types, anterior, inferior, posterior, and combination of these) and NSTEMIs. We used echo to document changes in cardiac output, which is an easily accessible tool in a resource-limited setting where more sophisticated devices, such as pulse contour analysis (PiCCO) or indicator dye dilution (LiDCO), aren’t available. We included patients on IABP and gave them an actual bolus of crystalloid to assess its impact on cardiac output.

Our results are limited by the relatively small number of patients, especially those on IABP. We had 21 such patients, and more data is required to assess volume responsiveness in such patients. Another limitation is that the dose of vasopressors was not quantified, and comparisons weren’t made between patients on mild, moderate, or severe doses of vasopressor to estimate responsiveness to fluid challenge in this cohort. Lastly, VTI is an echo based calculation and is subject to human errors.

## Conclusions

Fluid assessment is a key element in the management of patients with cardiogenic shock and is often the most unclear variable. We found that a simple bedside tool can be a strong predictor of response to fluids. Further studies that enroll larger numbers and assess the effects of fluid challenge directed management and their impact on time in shock, recovery from acute kidney injury, and days on assisted ventilation can further clarify the management of these patients.

## References

[1] Khalid L, Dhakam SH (2008). A review of cardiogenic shock in acute myocardial infarction. Curr Cardiol Rev.

[2] Sabatier C, Monge I, Maynar J, Ochagavia A (2012). Assessment of cardiovascular preload and response to volume expansion. (Article in Spanish). Med Intensiva.

[3] Monnet X, Marik PE, Teboul JL (2016). Prediction of fluid responsiveness: an update. Ann Intensive Care.

[4] van Diepen S, Katz JN, Albert NM (2017). Contemporary management of cardiogenic shock: a scientific statement from the American Heart Association. Circulation.

[5] Marik P, Bellomo R (2016). A rational approach to fluid therapy in sepsis. Br J Anaesth.

[6] Guerin L, Monnet X, Teboul JL (2013). Monitoring volume and fluid responsiveness: from static to dynamic indicators. Best Pract Res Clin Anaesthesiol.

[7] Myatra S, Prabu N, Divatia J, Monnet X, Kulkarni A, Teboul JL (2016). The changes in pulse pressure variation or stroke volume variation after a "tidal volume challenge" reliably predict fluid responsiveness during low tidal volume ventilation. Crit Care Med.

[8] Hernández G, Ospina-Tascón GA, Damiani LP (2019). Effect of a resuscitation strategy targeting peripheral perfusion status vs serum lactate levels on 28-day mortality among patients with septic shock: the ANDROMEDA-SHOCK randomized clinical trial. JAMA.

[9] Monnet X, Marik P, Teboul JL (2016). Passive leg raising for predicting fluid responsiveness: a systematic review and meta-analysis. Intensive Care Med.

[10] Aminiahidashti H, Shafiee S, Zamani Kiasari A, Sazgar M (2018). Applications of end-tidal carbon dioxide (ETCO2) monitoring in emergency department; a narrative review. Emerg (Tehran).

[11] Arango-Granados MC, Zarama Córdoba V, Castro Llanos AM, Bustamante Cristancho LA (2018). Evaluation of end-tidal carbon dioxide gradient as a predictor of volume responsiveness in spontaneously breathing healthy adults. Intensive Care Med Exp.

[12] Razi E, Moosavi GA, Omidi K, Khakpour Saebi A, Razi A (2012). Correlation of end-tidal carbon dioxide with arterial carbon dioxide in mechanically ventilated patients. Arch Trauma Res.

[13] Toupin F, Clairoux A, Deschamps A (2016). Assessment of fluid responsiveness with end-tidal carbon dioxide using a simplified passive leg raising maneuver: a prospective observational study. Can J Anaesth.

[14] Vahdatpour C, Collins D, Goldberg S (2019). Cardiogenic shock. J Am Heart Assoc.

[15] Omote K, Nagai T, Iwano H (2020). Left ventricular outflow tract velocity time integral in hospitalized heart failure with preserved ejection fraction. ESC Heart Fail.

[16] Vermeiren GL, Malbrain ML, Walpot JM (2015). Cardiac ultrasonography in the critical care setting: a practical approach to asses cardiac function and preload for the "non-cardiologist". Anaesthesiol Intensive Ther.

[17] Assadi F (2017). Passive leg raising: simple and reliable technique to prevent fluid overload in critically ill patients. Int J Prev Med.

[18] Steedman DJ, Robertson CE (1990). Measurement of end-tidal carbon dioxide concentration during cardiopulmonary resuscitation. Arch Emerg Med.

[19] Cantineau JP, Lambert Y, Merckx P, Reynaud P, Porte F, Bertrand C, Duvaldestin P (1996). End-tidal carbon dioxide during cardiopulmonary resuscitation in humans presenting mostly with asystole: a predictor of outcome. Crit Care Med.

[20] Grmec S, Klemen P (2001). Does the end-tidal carbon dioxide (EtCO2) concentration have prognostic value during out-of-hospital cardiac arrest?. Eur J Emerg Med.

[21] Khajebashi SH, Cholmaghani T, Nasr-Esfahani M (2021). Changes in end-tidal carbon dioxide (ETCO2) vs. changes in central venous oxygen saturation (ScvO2) and lactate clearance as a quantitative goal parameter in treatment of suspected septic shock patients. Adv J Emerg Med.

[22] Wiryana M, Sinardja I, GedeBudiarta I, Widnyana IM, Aryabiantara W, Paramasari AA (2017). Correlation of end tidal CO2 (ETCO2) level with hyperlactatemia in patient with hemodynamic disturbance. J Anesth Clin Res.

[23] Lim HS (2016). Cardiogenic shock: failure of oxygen delivery and oxygen utilization. Clin Cardiol.

[24] Jacquet-Lagrèze M, Baudin F, David JS, Fellahi JL, Hu PB, Lilot M, Piriou V (2016). End-tidal carbon dioxide variation after a 100- and a 500-ml fluid challenge to assess fluid responsiveness. Ann Intensive Care.

[25] Jozwiak M, Monnet X, Teboul JL (2018). Prediction of fluid responsiveness in ventilated patients. Ann Transl Med.

[26] Kreit JW (2019). Volume capnography in the intensive care unit: potential clinical applications. Ann Am Thorac Soc.

[27] Lakhal K, Nay MA, Kamel T, Lortat-Jacob B, Ehrmann S, Rozec B, Boulain T (2017). Change in end-tidal carbon dioxide outperforms other surrogates for change in cardiac output during fluid challenge. Br J Anaesth.

[28] Meneses LA, Carrillo-Torres O (2017). Cardiovascular response estimated by ETCO2 after leg-raising test. Rev Med Hosp Gen (Mex).

[29] Monge García MI, Gil Cano A, Gracia Romero M, Monterroso Pintado R, Pérez Madueño V, Díaz Monrové JC (2012). Non-invasive assessment of fluid responsiveness by changes in partial end-tidal CO2 pressure during a passive leg-raising maneuver. Ann Intensive Care.

